# Exploring Risks Transferred from Cloud-Based Information Systems: A Quantitative and Longitudinal Model

**DOI:** 10.3390/s18103488

**Published:** 2018-10-16

**Authors:** Wafa Bouaynaya, Hongbo Lyu, Zuopeng (Justin) Zhang

**Affiliations:** 1 Polytech Nantes, University of Nantes, 44200 Nantes, France; wafa.bouaynaya@univ-nantes.fr; 2 College of Logistics and E-Commerce, Zhejiang Wanli University, Ningbo 315100, China; 3 Coggin College of Business, University of North Florida, Jacksonville, FL 32224, USA

**Keywords:** cloud computing, IS risk, mathematical modeling, longitudinal study, organizational transformation

## Abstract

With the growing popularity of Internet of Things (IoT) and Cyber-Physical Systems (CPS), cloud- based systems have assumed a greater important role. However, there lacks formal approaches to modeling the risks transferred through information systems implemented in a cloud-based environment. This paper explores formal methods to quantify the risks associated with an information system and evaluate its variation throughout its implementation. Specifically, we study the risk variation through a quantitative and longitudinal model spanning from the launch of a cloud-based information systems project to its completion. In addition, we propose to redefine the risk estimation method to differentiate a mitigated risk from an unmitigated risk. This research makes valuable contributions by helping practitioners understand whether cloud computing presents a competitive advantage or a threat to the sustainability of a company.

## 1. Introduction

The growing popularity of Internet of Things (IoT) and Cyber-Physical Systems (CPS) has demanded more systems to be deployed in cloud-based environments in order to facilitate workflows and system functions in a large-scale network [1,2]. Developed from the convergence of several massive information processing technologies, cloud computing has become a paradigm in organizational transformation, particularly influencing small- and medium-sized businesses that use public cloud-based systems. The impact of cloud computing on the outsourcing process of information systems (IS) poses complex questions for market players in the digital economy. 

Issues of cloud computing such as loss of data control and ambiguity concerning its legal framework have revealed more and more of its disadvantages during its adoption. Many managers are suspicious of cloud computing when it comes to organizational, technological, and environmental risks [3,4]. Therefore, it is important to clearly understand the internal and external risks associated with adopting a cloud-based IS, particularly through a theoretical, quantitative, and longitudinal framework. 

Prior studies have developed frameworks of risk management to help the migration to a cloud-based system from various perspectives [3,5,6]. Nevertheless, these studies have not yet formally quantified and evaluated the risks within a real IS migration project. This study attempts to address this research gap by developing a conceptual model to quantify the risks resulted from a cloud computing context. Through mathematical modeling, our approach captures and investigates the variations of risks during the implementation of a cloud-based IS migration project.

Our research makes some significant contributions to the existing literature. First, we redefine the risk estimation formula by differentiating mitigated risks from unmitigated risks. Second, we suggest that the exposure to attenuated risk allows an interval of variation between maximum and minimum risk, which can serve as a reference for companies to limit an IS risk threshold. Third, we show that the variations of the internal and external risk are mutually dependent, obey a logic of geometric sequences and determined a general expression of this variation. Finally, we reject the hypothesis that the sum of internal and external risks is stable throughout a migration project to cloud computing, thus demonstrating the new risks exposed to companies from migrating to cloud-computing systems. 

This rest of the paper is structured as follows. The next section reviews prior literature on IS risks related to cloud computing adoption with the objective to identify an exhaustive list of such possible risks. Section 3 considers their variations over time and postulates hypotheses in line with our mathematical model. Section 4 presents a longitudinal case study of a cloud migration project to evaluate the proposed five hypotheses. A bivariate analysis is conducted between the theoretical model and the field results to confirm the model. The last section concludes the paper.

## 2. Prior Literature

Cloud computing has converged past technologies such as virtualization, grid computing, and broadband networks [7], which has significantly changed the existing standards in terms of the growth of IT resources and their decreased costs [3,8]. Thus, information systems management has become less expensive [9].

Research on cloud computing adoptions has been largely inspired from IT adoption theories such as the theory of planned behavior (TPB) and the technology acceptance model (TAM) or their extensions [10,11,12]. They considered that the consumer is at the center of the analysis and suggest that perceived utility and ease of use determine the choice of cloud computing adoptions. Ease of use, universal access to files, and availability of groupware positively influence a consumer’s attitude towards cloud computing [12]. However, their contributions do not eliminate privacy and security concerns [13,14,15]. According to Li and Chang [11], security, privacy, and reversibility accounted for nearly 33% of the perceived risk variance (behavior’s influence factor).

Outsourced from an expert provider, cloud computing was initially designed to solve a security problem by reducing an organization’s number of servers and subsequent network infrastructure size [11]. But this option quickly leads to a feeling of uncertainty following the controversy launched by Snowden in 2013 [7]. Because it is risky to implement information technology [16,17], migration to SaaS-based IS is typically slow and cautious, especially for companies with the capacity to invest in IT infrastructure [4].

Many information technology projects fail due to various reasons [18,19]. With productivity improvements being delayed [20], managers find it difficult to see the usefulness of the proposed IS projects. In addition, the disadvantages of migrating to cloud computing [21,22] appear to be connected to the process of data outsourcing.

Lack of confidence in cloud service providers is one of the obstacles to rapid adoption [4,23]. Stieninger et al. [21] explained that trust was strongly correlated with security and its perception. They identified four key elements that guided the cloud computing adoption: Data security, trust in the service provider, contractual agreements and geographical location. Other authors [4,24,25] add the possibility of transferring data and programs from one provider to another. According to Armbrust et al. [22], services’ non-reversibility resulting from data confinement is one of the limitations of the continuous growth of cloud computing. They call for a standardization of cloud computing APIs for interoperability between different providers. Similarly, Troshani et al. [3] suggested a Cloud Computing Risk Management Framework that subdivides threats into three main axes: A technology, an organizational, and an environmental axe. Their work focuses on the risks associated with cloud computing that can influence its adoption.

In summary, prior research has evaluated the dependencies among different factors as well as identifying the risks in a cloud computing framework. However, existing studies have not systematically quantified the risks associated with a cloud-based IS and assessed the variations of risks before and after its adoption. Therefore, we propose to evaluate such risks through a quantitative and longitudinal model which spans the entire life cycle of a cloud-based IS project from its launch to completion. Our goal is to better understand whether migrating to a cloud computing system can bring competitive advantages or pose a threat to a company.

## 3. Research Model

Risk is defined by a triggering event (risk factor) and the scope of the affected component. It is expressed through the probability of the scenario occurrence and the impact severity on the component. The risk exposure measure proposed by Boehm [26] in software engineering is suggested as risk exposure: $E\left(R_{i}\right)=P\left(R_{i}\right)\times I\left(R_{i}\right)$, where $P\left(R_{i}\right)$ denotes the scenario probability and $I\left(R_{i}\right)$ represents the severity of impact of the risks. In finance, risk is defined as results’ distribution variance [27,28] and the estimation of this occurrence probability is normally based on historical data [29]. Other fields have also attempted to subjectively estimate the probability of the risk factors [30].

Some characteristics of cloud risks intersect with those in supply chain networks or the financial sector [18]. For instance, in a cloud context, customers may be exposed to a risk of default from their cloud providers [3,29]. According to Cloud Harmony’s performance indicators, in 2014 Microsoft Azure scored 103 breakdowns that affected a large number of its customers for a total of 42.94 h of downtime. Therefore, we can estimate the probability of the downtime risk of a cloud service based on vendor history. For other types of risks such as environmental or malicious accidents, the estimate can only be based on subjective criteria. The subjectivity of risk management methods is still criticized [30,31,32]. Some authors found that several frameworks are not scientific or do not adequately address the system risks. Moreover, these methods are concerned with their focus on a technical aspect by considering the social aspects as a simple obstacle to overcome [31,33].

Current risk management methods can be divided into three generations [30]: The first two generations focus on the general requirements for systemic risks based on good practices or checklists, whereas the third generation exceeds the application of generic standards by integrating organizational requirements such as the human component [31,34].

Although risks often result from human behavior directly or indirectly, the human component has long been neglected by systemic risks studies [35,36]. An interpretive perspective within risk management is called for because it would lead to a multidimensional view [37] that goes beyond the simplistic explanations provided by the functionalist paradigm.

Indeed, the risk estimate is evaluated without considering the reduction factors that include the human component like preventive, deterrent, palliative, and containment measures. Therefore, it is important to distinguish between mitigated and unmitigated risks and to redefine exposure to risks by taking these measures into account.

Preventive and dissuasive measures act on the factors that reduce the event occurrence probability, while palliative and containment measures act on the impact reduction factors on the component. Consequently, we suggest that the exposure to attenuated risk is defined as follows:

E(RiA) = [P(Ri) − M(Pr,Ds)] × [I(Ri) − M(Pl,Cn)](1)

where M(Pr,Ds) ≠ 0 and M(Pl,Cn) ≠ 0 and the notations used in the formula are shown in Table 1. 

We argue that the risk exposure formula suggested by Boehm [26] is only applicable to estimate the exposure to unmitigated risk:

If M(Pr,Ds) = 0 and M(Pl,Cn) = 0 <=> E(RiA) = E(RiNA)(2)

Therefore, we note the estimate of the non-attenuated risk by E(RiNA) and we propose a first hypothesis:

**Hypothesis 1** **(H1).** Information System risk quantification is included in an interval [RiA; RiNA], where RiA = The estimation of ed risks, and RiNA = The estimation of the mitigated risks.

To quantify IT risks, we need to understand IT governance methodologies. IT governance has gained significant research interest since the application of US Sarbanes-Oxley or HIPPA laws to mitigate IT risks [38]. Although no governance model covers all possible controls, each model responds to some requirements that affect either procedures, objectives, or scope of coverage.

IT governance in a cloud computing context requires a new definition of organizational policies. It must explicitly describe roles and responsibilities for the management of technologies, business processes, and applications. Indeed, the cloud computing adoption does not change the objectives set by IT governance standards. However, it introduces to cloud providers a new relational element [8] that must be included in IT governance deployment. So, traditional IT governance models (COSO, CobiT, ENISA, ITIL and ISO) are not altered by implementing cloud solutions, but they must be adapted to such a new context.

In 2011, the Information Systems Audit and Control Association (ISACA) tried to adapt the Cobit repository to a cloud context. They suggested a new publication of IT governance titled “IT control objectives for cloud computing”. The study described the technological and organizational requirements of setting up a repository including cloud computing systems. In addition, COSO has submitted an enterprise risk management framework (ERM framework) for the governance of cloud computing through seven guidelines, which can be tailored to business process, deployment models, and cloud service models, and can also be merged with the Cloud Cube Model suggested by the Open Group to include the four characteristic dimensions of the service instead of the cloud options

ISO has also published two new standards in adequacy with the requirements of cloud computing: ISO/IEC 27017 and ISO/IEC 27018. The first provides guidelines for the implementation of information security controls for cloud services in addition to the initial guide defined by ISO 27002. The second encompasses best practices for protecting of personal identifiable information (PII) in public cloud computing. The 2700× series of ISO/IEC standards are often associated with the harmonized method of risk analysis (MEHARI), which is developed by CLUSIF. Through personalized measures, MEHARI suggests analyzing corporate business challenges to reduce risk exposure. The method reached its sixth version and shows an advanced maturity in risk management.

To develop our model, we retain some suggestions in the MEHARI 2010 version (see Appendix A). First, we construct a comprehensive list of IS risks based on the MEHARI 2010 event typology. Then, we add to the list the five incidents that can arise in a cloud computing context and finally we integrate risks related to project management [18]. Secondly, we develop a matrix in which rows represent the list of event triggers of risk and columns the temporal phases of a cloud computing project. The temporal definition of actions is a key element in studying the phenomenon course [39]. So, it is important to break down the timeframe and define appropriate periods to match the project evolution. We consider time as a social construct and retain the organizational transformation model suggested by Besson and Rowe [40] to define the four-phase migration project: Uprooting, exploration and construction of the new solution, stabilization and the institutionalization of the new solution, and optimization of new routines. Finally, by applying the formula E(RiA), we specify a type for each event and each phase (external, internal, or both at the same time), a maximum estimate (i.e., the risk is unmitigated), and a minimum estimate (i.e., the risk is mitigated). Appendix B shows the precise values of all the parameters and Appendix C summarizes the measures of theoretical risk estimation with respect to the type and σ E(Ri).

Alter and Sherer [41] distinguish between a permanent and a temporary risk, but we consider that any risk is a temporary risk since its probability or impact may be zero at a specific time t. In addition, we add the estimates of events to each organizational transformation phase to quantify the evolution of internal risks and external risks. If a risk is both external and internal, we divide its estimate by two. For each phase, we obtain two values for each type of risk: A minimum value (attenuated risk) and a maximum value (non-attenuated risk). These values make it possible to define a variation interval [RiA; RiNA]. There is a gradual increase of 1/2 of the external risks and a reduction of 1/3 of the internal risks. It is also important to note that the internal risk represents approximately 75% of the total risk at the beginning of a cloud computing project and the external risk represents 25% (see Table 2). These probabilities are reversed at the end of the project. Therefore, we propose the second hypothesis as follows.

**Hypothesis 2** **(H2).** *The internal IS risk represents 3/4 of the total risk before launching a project to migrate to the cloud, but 1/4 of the project’s completion.*

**Figure 1:**
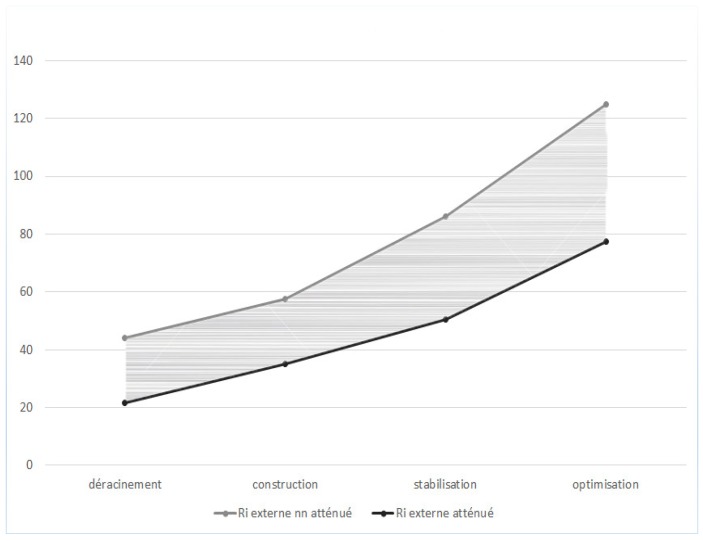
Evolution of external risks according to time.

**Figure 2:**
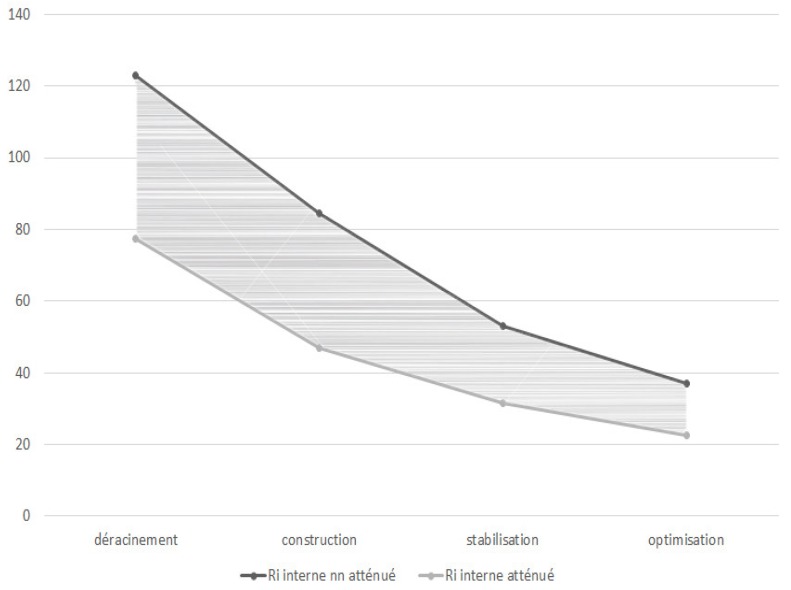
Evolution of internal risks according to time.

We observe that the variation of internal risks and external risks over time is a geometric sequence of respective reasons 2/3 and 3/2 (see Figure 1 and Figure 2). So, we can propose a new hypothesis and express the sequences of internal risk (Rin) and external risk (Rex) as:

Rint = 2/3 × Rin _t−1_ and Rext = 3/2 × Rex _t−1_ (3)

The number of intervals between the phases (4 points) is 3. Therefore, we induce the geometric sequence increases or decreases per unit of time. To generalize:

when n denotes the number of intervals (or unit of time) and n > 1,

Rint = (n − 1/n) × Rin_t−1_ and Rex_t_ = (n/n − 1) × Rex_t−1_ (4)

According to a numerical analysis, we note the expression of the internal risk and the external risk at a time t as:

Rin(t) = (n − 1/n)^t^ × Rin(0) and Rex(t) = (n/n − 1)^t^ × Rex(0) (5)

**Figure 3:**
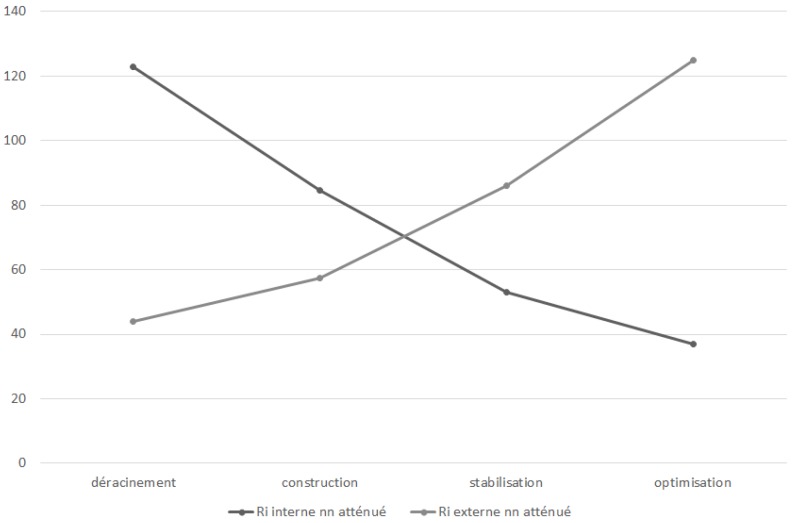
Unmitigated risk transfer model.

**Figure 4:**
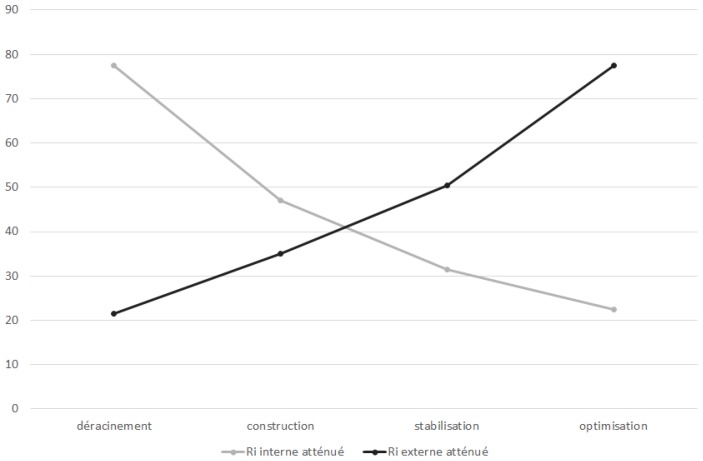
Mitigated risk transfer model.

Hence, we next propose the third and fourth hypotheses (see Figure 3 and Figure 4).

**Hypothesis 3** **(H3).** *The internal risk decreases by 1/3 from one phase to the next within a four-stage cloud computing project.*

**Hypothesis 4** **(H4).** *The external risk increases by 1/2 from one phase to the next within a four-stage cloud migration project.*

We induce that cloud computing does not expose the company to new risks. However, with cloud computing, risks transfer from the inside to the outside. A cloud computing choice is in fact an agreement of IS risk outsourcing to cloud providers. Therefore, we propose the following final hypothesis:

**Hypothesis 5** **(H5).** *The sum of internal risks and external risks is always the same throughout the four stages of a cloud computing project.*

## 4. Empirical Study

### 4.1. Research Methodology

We apply a qualitative research methodology by focusing on a longitudinal case study of a cloud computing project. The longitudinal approach has a confirmatory character for our deductive approach. It precisely defines the phases of a project, so we can measure the risks at the appropriate time. Its objective is to understand the outcome of a phenomenon through the definition of three key elements: Context, actions, and the temporal interconnection between actions [39].

First, we identified around ten French SMEs offering PaaS and (or) IaaS cloud services that could be interested in our work. The selection criteria were the size of the company, the geographical accessibility of the servers, and the simplicity of the communication with their potential customers. Two of them showed interest and engaged in the study process. However, one field research had to be terminated because of the contradictions between data provided and the data collected. Typically, managers are uncomfortable when asked to communicate on IS security issues, so their participation rates in studies do not exceed 1.8% [42].

To develop our remaining case study in the second company, several of its customers were contacted. The cloud provider was not in direct contact with them and lacked data to assess the risks in the first phase. The selection criteria were their sizes, their sectors of activities, and the nature of the cloud computing project.

First, our empirical study was based on the processing of primary data through several semi-directive interviews, with the technical director and the IT security manager of the cloud provider to contextualize the project and define the major purposes of our empirical research. Second, we conducted another semi-structured interview with the customer’s CIO. Then we organized a working session at the local cloud provider with the IT security manager. Another work session was also planned with the client’s CIO. They were conducted as directional interviews so that the IT security manager and the CIO could correctly estimate the probability and impact of each event. The objective was to quantify the risks with the best precision through the evaluation grid that we previously suggested.

The research proposal and evaluation grids were sent to the interviewees before the interviews so that they could assess the research project in advance. In the meantime, we had exchanged information by telephone and e-mail to meet our expectations. We had also used several sources to collect secondary data such as press releases, data available on the Internet, and the configuration documents offered in free access on GitHub. In addition, we watched several videos describing the datacenter. Excluding guided tours during the Heritage Days, access to the site was restricted for security reasons. Therefore, it was not possible to evaluate the risk management measures except through the video and photo footage suggested. 

The longitudinal study lasted approximately 5 months. Finally, we were able to compare the risk measures taken by the cloud provider’s CISO and the client’s CIO with our comments.

### 4.2. A Longitudinal Study

The first step in a longitudinal study is to complete a monograph of the process studied [43]. The studied process is a transfer of IS risks during a migration project to the cloud. It is important to describe in detail the sequence of events and thus to understand the temporal interconnections between these events. 

The cloud computing projects used in companies generally correspond to a support use. Few companies take the risk of outsourcing core activities to a hosted service. However, our case study is different because our empirical study is not a study of auxiliary activities but the follow-through of the core business migration. 

We studied a trading platform initially developed in-house by a French start-up in 2010. Its objective was to offer a communication tool through social networks or websites to companies who wish to create a direct link with their permanent or potential customers. Thanks to this platform, the synchronization of communications between companies and customers will, in the long term, increase user satisfaction and loyalty.

Currently, the start-up company employs 200 people (see the client features in Table 3). In 2015, it rationalized its offer of customer intermediation and acquired another French start-up offering a social network monitoring service. The platform also offered a connector to synchronize its tools with the Salesforce CRM solution.

In 2010, before its implementation of cloud computing projects, the platform prototype development lasted several months. The start-up was one of the cloud provider’s first customers. This initial internal development took a relatively long time compared to the duration needed to host the solution within the cloud provider’s data-centers. However, we prefer a social construction chronology to a standard time one [44]. We also retain, as we did in our theoretical proposition, the transformation organizational structure model suggested by Besson and Rowe [40].

The process studied must be subdivided into several phases that fit a relatively homogeneous set [45]. Internal development then corresponds to the phase of uprooting or “revolution” [40]. We break-down the implementation phase into two phases: A phase of construction which begins with the first set-up operations and a stabilization phase at the end of these operations and the completion of the stabilization tests. A final step, the optimization phase, is defined by the launch of the product to the general public during the year 2011.

The empirical study was mainly carried out at the cloud computing provider’s premises. Our exchanges with the customer CIO focused on risk measures during the uprooting phase. We also validated the internal risk measures suggested by the cloud provider. 

The cloud provider is a French company, created in 2010 and located in the same region as its customer. It offers a Platform as a Service (PAAS) cloud solution supporting the programming languages: PHP, Java, Ruby and Scala. Its pricing system is based on energy costs automatically adjusting to potential load increases (see the cloud provider’s features in Table 4).

The PaaS provider started its services based on a partnership with a French telephone company that has five data centers based in Paris. In 2014, it launched another data-center in Canada to target the US market. The data of French customers is always hosted in France.

Although the cloud provider stated that French data-centers were Tier IV certified, we cloud only identify one Tier III certified data-center. There remains, however, a high security guarantee. A Tier III data-center offers 99.98% availability within 1.6 h of outage per year. Its configuration provides maintainability of all data-center components without impact on service continuity. Note that it has a partial redundancy of N + 1 in contrast to Tier IV which has a 2N + 1 redundancy.

The four-tier certification is issued by a US private organization, the uptime institute, based on design documents and building construction. The institute is limited to climate and electrical redundancies and does not take into account data replication software or clustered servers. Therefore, the security guarantee is partial and costs a hundred thousand Euros per data-center.

Many data-center manufacturers have abandoned the certification process to self-proclaim as Tier III + or Tier IV. They are based on the 2N + 1 redundancy model or prefer to apply a standard of the ISO 270xx series. ISO 27017 and ISO 27018 offer specific guides to cloud computing and guarantee a security policy for application services. In France there are only three data-centers certified partly third III or IV.

On the application side, the cloud provider has opted for hypervisor-based virtualization. Their customers’ applications are thus partitioned to their own virtual machines. They guarantee a total isolation of each application distribution. This strategic choice is driven by security reasons. Indeed, virtualization techniques can be categorized into two major families: Container virtualization and hypervisor-based virtualization. Although container virtualization offers a lighter, more powerful virtual environment [46], it poses a problem of isolation between applications and the host kernel [47]. It exposes hosted data to an intrusion risk.

We have chosen to organize the risk transfer process in a matrix shape so as to simplify taking measures for CIOs. The four phases of the project are displayed in columns and the different events triggering an IS risk in rows. The narrative text is spread over several pages and does not facilitate the comparison of one or more variables over several periods. Such narratives are criticized for structuring a longitudinal study [48]. Therefore, we used the chronological matrix, expressing at each phase a type of risk (external, internal, or both), its probability, and its impact.

### 4.3. Results and Implications

Our empirical results indicate that the measured external and internal risks vary within the range [20, 100], which confirms the first hypothesis (see Table 5 for results in summary and Appendix D for results in in details).

The first contribution of our work suggests a definition of a risk interval for a cloud project migration. This proposal is also useful for quantifying IS risks in a global way within an organization. Exceeding the indicated threshold may alert the company to a possible problem in its risk management approach.

**Figure 5:**
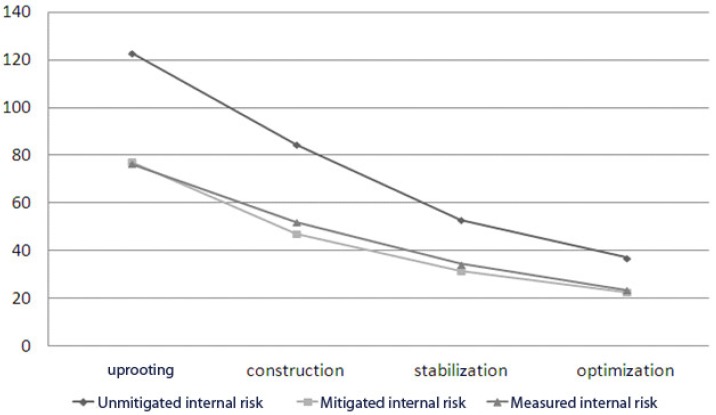
Measured internal risk variation.

**Figure 6:**
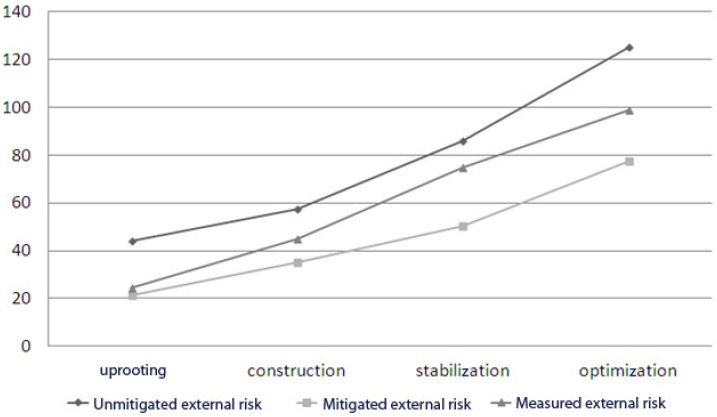
Measured external risk variation.

We next perform a covariance analyzes, including all the measured variables and expected theoretical variables, to validate the rest of the hypotheses. The objective is to model the homogeneity between the measured values and the theoretical values. Figure 5 and Figure 6 visually present the results generated.

First, we drew regression curves for changes in measured internal and external risks. Hence, it is possible to estimate the first two theoretical values Rex(1) and Rin(1). From applying the two formulas Rex(t) and Rin(t) suggested in the theoretical framework, we can generate these two new sets of theoretical values. Therefore, we define the regression line of the external risks’ variation as

Yex = 25.35 × Xex − 2.5,(6)

and the regression line of the internal risks variation with respect to time as:

Yin = (−17.65) × Xin + 90.75.(7)

The expected values of Rex(1) and Rin(1) are 23 and 73. We can thus construct two sets of expected theoretical values and then compare the distributions of internal and external risks to confirm our model (see Table 6 for the theoretical risk variation).

Finally, a bivariate analysis is carried out to define the dependence between the theoretical model and the results obtained. The distribution parameters used in this approach are the covariance, the correlation coefficient, and the coefficient of determination. The coefficient of determination is an indicator that allows approving the model quality through the adequacy between the latter and the observed data. Therefore, it will be of great value for validation of the hypotheses.

The correlation coefficient and coefficient of determination R2 measurements are 0.999 and 0.999 for the internal risk model and they are 0.988 and 0.976 for the external risk model. We can therefore confirm both hypotheses H3 and H4.

**Figure 7:**
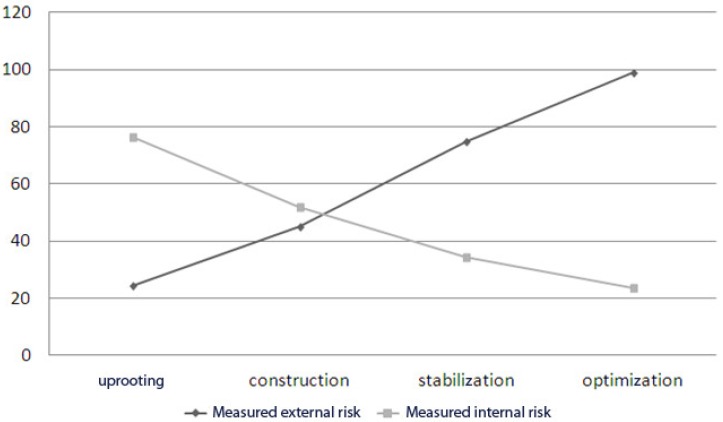
Measured IT risk transfer.

However, the second hypothesis H2 remains rejected since the internal risk represents 3/4 of the total risk before the launch of the project but not the 1/4 at the end. Indeed, the coefficient of variation of external risks (0.466) is higher than that of internal risks (0.429). The rapid increase in external risks has shifted the balance established to reduce internal risks to 1/5 of total risks at the end of project (see Figure 7). So, it is possible to confirm that the internal risk is significantly higher than the external risk before the launch of the cloud project. This dispersion is reversed at the end of the project without specifying the distributions. The reality can be known only in a probabilistic way and the verification is not probative [49].

The last hypothesis implies that the variable “sum of risks” is independent of the variable “time”. Consequently, the covariance value of the two series tends to zero. Although the covariance, equal to 9.87, is relatively small, it cannot validate the hypothesis. An approximate increase of 1/5 of the total risk is noticed at the completion of cloud computing project. The hypothesis H5 is to be rejected, and it is, therefore, conceivable that cloud computing exposes the company to new risks. Other case studies should be planned to confirm or reject this hypothesis. Only the refutation of the hypotheses is conclusive [49].

## 5. Conclusions

Our work indicates a redefinition of the risk estimation formula suggested by Boehm [26], including probability reduction factors and impact reduction factors. We distinguished between mitigated and unmitigated risks. The factors of probability reduction are conditioned by preventive and dissuasive measures, whereas the factors of the impact reduction are conditioned by palliative and containment measures. Therefore we suggest that the exposure to attenuated risk is defined as: E(RiA) = [P(Ri) − M(Pr,Ds)] × [I(Ri) − M(Pl,Cn)], which allows an interval of variation between maximum risk and minimum risk. Apart from its contribution to the validation of the theoretical model during the empirical study, this interval can serve as a reference for several companies to limit an IS risk threshold.

Our positivist approach also revealed a transfer of IS risk from the inside to the outside during a cloud computing project through a longitudinal mathematical model. We have shown that the variation of the internal risk and external risk are mutually dependent and obeys a logic of geometric sequences of respective reasons 2/3 and 3/2 for a four-phase organizational transformation model (three intervals). Therefore, we have determined a general expression of this variation: Rin(t) = (n − 1/n)t × Rin(1) and Rex(t) = (n/n − 1)t × Rex(1). 

On the other hand, we rejected the hypothesis that the sum of internal and external risks is stable throughout a migration project to cloud computing. An approximate increase of 1/5 of the total risk is noticed at the completion of the cloud computing project. It is, therefore, conceivable that cloud computing exposes the company to new risks. 

## Figures and Tables

**Table 1:** Summary of Notations.

Notation	Meaning
RiA	Attenuated risk
P(Ri)	Scenario probability
I(Ri)	Severity of impact
M(Pr)	Preventive measures
M(Ds)	Dissuasive measures
M(Pl)	Palliative measures
M(Cn)	Containment measures

**Table 2:** Total Risks for Each Phase.

	Uprooting	Construction	Stabilization	Optimization
Σ Unmitigated internal risks: R1in	123	84.5	53	37
Σ Mitigated internal risks: R2in	77.5	47	31.5	22.5
Σ Unmitigated external risks: R1ex	44	57.5	86	125
Σ Mitigated external risks: R2ex	21.5	35	50.5	77.5

**Table 3:** Client Features.

Creation Date	2010
Legal Form	joint stock company
Capitalization (2015)	14 million Euros
Turnover (2014)	4.2 million Euros
Number of staff	200

**Table 4:** Cloud Provider Features.

Creation Date	2010
Legal Form	joint stock company
Share capital	18,000 euro
Turnover (2012)	84,000 euro
Number of employees	10

**Table 5:** Risk variation measured.

	Uprooting	Construction	Stabilization	Optimization
External Risk	24.5	45	75	99
Internal Risk	76.5	52	34.5	23.5

**Table 6:** Theoretical risk variation.

	Uprooting	Construction	Stabilization	Optimization
External Risk	23	35	53	79
Internal Risk	73	49	33	22

## Appendix A. The MEHARI 2010 Framework

**Table A1:** MEHARI 2010 Standard Scale of Impact Level.

4	level 4	Vital
3	level 3	Very serious
2	level 2	Important
1	level 1	Not significant

**Table A2:** MEHARI 2010 Standard Scale of Potential Level.

4	level 4	Very probable
3	level 3	Probable
2	level 2	Improbable
1	level 1	Very improbable

**Table A3:** Effectiveness of MEHARI 2010 deterrence measures.

level 4	The deterrent effect is very important
level 3	The deterrent effect is important
level 2	The deterrent effect is medium
level 1	The deterrent effect is very low

**Table A4:** Effectiveness of MEHARI 2010 preventive measures.

level 4	The preventive effect is very important
level 3	The preventive effect is important
level 2	The preventive effect is medium
level 1	The preventive effect is very low

**Table A5:** Effectiveness of MEHARI 2010 Containment Measures.

level 4	The confinement and limitation effect of direct consequences is very important
level 3	The confinement and limitation effect of direct consequences is important
level 2	The confinement and limitation effect of direct consequences is medium
level 1	The confinement and limitation effect of direct consequences is very low

**Table A6:** Effectiveness of MEHARI 2010 palliative measures.

level 4	The effect of limiting indirect consequences is very important
level 3	The effect of limiting indirect consequences is important
level 2	The effect of limiting indirect consequences is medium
level 1	The effect of limiting indirect consequences is very low

## Appendix B. Measures of Theoretical Risk Estimation—General Framework

**Table A7:** Measures of Theoretical Risk Estimation—General Framework.

Code	Uprooting				Construction				Stabilization				Optimization			
	P(Ri)	I(Ri)	*M(Pr,Ds)*	*M(Pl,Cn)*	P(Ri)	I(Ri)	*M(Pr,Ds)*	*M(Pl,Cn)*	P(Ri)	I(Ri)	*M(Pr,Ds)*	*M(Pl,Cn)*	P(Ri)	I(Ri)	*M(Pr,Ds)*	*M(Pl,Cn)*
AB.P.1	2	1	1	0	2	1	1	0	2	1	1	0	2	1	1	0
AB.P.2	3	2	1	0	3	2	1	0	2	2	1	0	2	2	1	0
AB.S.1	2	3	1	0	1	1	0	0	1	1	0	0	2	3	1	0
AB.S.2	2	1	0	0	1	1	0	0	1	1	0	0	2	3	1	0
AB.S.3	1	2	0	0	1	1	0	0	1	1	0	0	1	1	0	0
AB.S.4	4	2	1	0	0	0	0	0	0	0	0	0	1	3	0	1
AB.S.5	4	2	1	0	0	0	0	0	0	0	0	0	1	3	0	1
AC.E.1	1	3	0	2	1	3	0	1	1	3	0	1	1	3	0	2
AC.E.2	2	4	0	2	1	3	0	1	1	3	0	1	1	4	0	2
AC.E.3	2	4	0	2	1	3	0	1	1	3	0	1	2	4	0	2
AC.M.1	2	1	1	0	2	2	1	0	1	2	0	1	1	2	0	1
AC.M.2	1	1	0	0	1	1	0	0	1	1	0	0	1	1	0	0
AV.P.1	3	1	1	0	2	1	1	0	1	1	0	0	1	1	0	0
ER.L.1	1	2	0	0	2	1	0	0	2	2	0	1	1	3	0	1
ER.P.1	3	1	1	0	3	1	1	0	2	1	1	0	2	1	1	0
ER.P.2	3	2	1	0	3	2	1	0	2	2	1	0	2	2	1	0
ER.P.3	4	1	1	0	4	1	1	0	3	1	1	0	3	1	1	0
IC.E.1	3	1	1	0	2	2	1	0	1	2	0	0	1	2	0	0
IC.E.2	2	2	0	1	2	2	0	1	1	2	0	1	1	2	0	1
IC.E.3	2	2	0	1	2	2	0	1	1	2	0	1	1	2	0	1
IC.E.4	1	1	0	0	1	1	0	0	1	1	0	0	1	1	0	0
IF.L.1	2	1	1	0	2	1	1	0	2	1	1	0	2	1	1	0
IF.L.2	2	2	0	1	2	2	0	1	2	2	0	1	1	3	0	1
IF.L.3	3	2	1	1	2	2	1	1	1	2	0	1	1	3	0	1
IF.L.4	4	2	2	0	3	2	2	0	2	3	1	1	1	3	0	1
MA.L.1	1	2	0	0	1	1	0	0	1	2	0	0	2	3	1	1
MA.L.2	1	2	0	0	1	1	0	0	1	2	0	1	1	3	0	1
MA.L.3	1	2	0	0	1	2	0	0	1	3	0	1	1	3	0	1
MA.L.4	2	2	0	1	2	2	0	1	1	3	0	1	1	3	0	1
MA.L.5	1	3	0	1	1	3	0	1	1	3	0	1	1	3	0	1
MA.L.6	1	3	0	1	1	3	0	1	1	3	0	1	1	3	0	1
MA.L.7	1	2	0	1	1	2	0	1	1	2	0	1	1	2	0	1
MA.L.8	1	2	0	0	1	2	0	0	1	3	0	0	1	3	0	0
MA.L.9	2	3	1	0	2	3	1	0	1	4	0	1	1	4	0	0
MA.L.10	3	3	2	0	3	3	2	0	3	3	2	0	2	3	1	0
MA.P.1	1	2	0	1	1	3	0	1	1	3	0	1	1	3	0	1
MA.P.2	1	3	0	1	1	3	0	1	1	3	0	1	1	3	0	1
MA.P.3	1	3	0	1	1	2	0	1	1	2	0	1	1	2	0	1
MA.P.4	2	2	1	1	2	2	1	1	2	2	1	1	2	2	1	1
PR.N.1	2	2	1	1	2	2	1	1	1	2	0	1	1	2	0	1
PR.N.2	2	2	0	0	1	2	0	0	1	2	0	0	1	2	0	0
PR.N.3	2	2	1	0	2	2	1	0	1	2	0	0	1	2	0	0
PR.N.4	1	2	0	1	1	2	0	1	1	2	0	1	1	2	0	1
IC.C.1	0	0	0	0	2	3	0	1	1	3	0	1	1	3	0	1
IC.C.2	0	0	0	0	0	0	0	0	2	2	1	0	1	2	0	0
IC.C.3	0	0	0	0	0	0	0	0	4	2	2	0	4	3	2	0
IC.C.4	0	0	0	0	0	0	0	0	0	0	0	0	4	2	1	0
IC.C.5	0	0	0	0	0	0	0	0	0	0	0	0	4	2	1	0
RS.P.1	0	0	0	0	2	1	1	0	2	2	1	0	0	0	0	0
RS.P.2	0	0	0	0	2	1	1	0	2	2	1	0	0	0	0	0
RS.P.3	0	0	0	0	2	1	1	0	2	2	1	0	0	0	0	0
RS.P.4	0	0	0	0	1	1	0	0	1	1	0	0	0	0	0	0
RS.P.5	0	0	0	0	1	1	0	0	1	1	0	0	0	0	0	0

## Appendix C. Measures of Theoretical Risk Estimation—Type and σ E(Ri)

**Table A8:** Measures of Theoretical Risk Estimation—Type and σ E(Ri).

Family Type	Code	Event Description	Uprooting		Construction		Stabilization		Optimization	
			Type	σ E(Ri)	Type	σ E(Ri)	Type	σ E(Ri)	Type	σ E(Ri)
Absence of personnel due to an accident	AB.P	Absence of personnel from partner	ex	2−1	ex	2−1	ex	2−1	ex	2−1
		Absence of internal personnel	in	6−4	in	6−4	in	4−2	in	4−2
Accidental lack or unavailability of service	AB.S	Absence of service: Power supply	in	6−3	ex	1	ex	1	ex	6−3
		Absence of service: air conditioner	in	2	ex	1	ex	1	ex	6−3
		Absence of service: impossibility to have access to the premises	in	2	ex	1	ex	1	ex	3−1
		Absence or impossibility of application software maintenance	in	8−6	in	0	ex	0	ex	3−2
		Absence or impossibility of information system maintenance	in	8−6	in	0	ex	0	ex	3−2
Environmental serious accident	AC.E	Lightning	ex	3−1	ex	3−2	ex	3−2	ex	3−1
		Fire	ex	8−4	ex	3−2	ex	3−2	ex	4−2
		Flooding	ex	8−4	ex	3−2	ex	3−2	ex	8−4
Hardware Accident	AC.M	Equipment breakdown	in	2−1	ex	4−2	ex	2−1	ex	2−1
		Accessory equipment breakdown	in	1	in	1	in	1	in	1
Voluntary absence of staff	AV.P	Social conflict with strike	in	3−2	in	2−1	in	1	in	1
Design error	ER.L	Software blocking or malfunction due to a design or programming error (in-house software)	in	2	ex	2−1	ex	4−2	ex	3−2
Hardware error or behavioral error by personnel	ER.P	Lost or forgotten document or media	in	3−2	ex/in	3−2	ex/in	2−1	in	2−1
		Error of operation or non compliance of a procedure	in	6−4	ex/in	6−4	ex/in	4−2	in	4−2
		Typing or data entry error	in	4−3	in	4−3	in	3−2	in	3−2
Incident due to environment	IC.E	Damage due to aging	in	3−2	in	4−2	ex/in	2	ex	2
		Water damage	in	4−2	in	4−2	ex/in	2−1	ex	2−1
		Electrical boosting or over load	in	4−2	in	4−2	ex/in	2−1	ex	2−1
		Pollution damage	in	1	in	1	ex/in	1	ex	1
Logical or functional incident	IF.L	Production incident	in	2−1	in	2−1	in	2−1	in	2−1
		Software blocking or malfunction (information system or software package)	in	4−2	in	4−2	ex	4−2	ex	3−2
		Saturation due to an external cause (worm)	ex	6−2	ex	4−1	ex	2−1	ex	3−2
		Virus	ex	8−4	ex	6−2	ex	6−2	ex	3−2
Malevolent action (logical or functional)	MA.L	Deliberate blocking of accounts	ex/in	2	ex	1	ex	2	ex	6−2
		Deliberate erasure or massive pollution of system configurations	in	2	ex	1	ex	2−1	ex	3−2
		Deliberate erasure of files, data bases or media	in	2	ex/in	2	ex	3−2	ex	3−2
		Electromagnetic pick up	ex/in	4−2	ex	4−2	ex	3−2	ex	3−2
		Deliberate corruption of data or functions	in	3−2	in	3−2	ex/in	3−2	ex/in	3−2
		Forging of messages or data	in	3−2	ex/in	3−2	ex/in	3−2	ex/in	3−2
		Fraudulent replay of transaction	in	2−1	ex	2−1	ex	2−1	ex	2−1
		Deliberate saturation of IT equipments or networks	ex/in	2	ex	2	ex	3	ex	3
		Deliberate total erasure of files and backups	in	6−3	in	6−3	ex	4−3	ex	4
		Diversion of files or data (tele-load or copy)	in	9−3	in	9−3	ex/in	9−3	ex/in	6−3
Malevolent action (physical)	MA.P	Tampering or falsification of equipment	in	2−1	ex	3−2	ex	3−2	ex	3−2
		Terrorism	ex/in	3−2	ex/in	3−2	ex/in	3−2	ex/in	3−2
		Vandalism or hooliganism	ex/in	3−2	ex/in	2−1	ex/in	2−1	ex/in	2−1
		Theft of physical asset	ex/in	4−1	ex/in	4−1	ex/in	4−1	ex/in	4−1
Non compliance to procedures	PR.N	Inadequate procedures	in	4−1	in	4−1	in	2−1	in	2−1
		Procedures not applied due to lack of resource or means	in	4	in	2	in	2	in	2
		Procedures not applied due to ignorance	in	4−2	in	4−2	in	2	in	2
		Procedures not applied deliberately	in	2−1	in	2−1	in	2−1	in	2−1
Cloud Computing Incident	IC.C	Altering data transferred to the cloud	in	0	ex/in	6−4	ex/in	3−2	ex/in	3−2
		Denial of Service	ex	0	ex	0	ex	4−2	ex	2
		Unauthorized access to data by a third party (supplier’s personnel, government access by conflict of laws, etc.)	ex	0	ex	0	ex	8−4	ex	12−6
		Data backup by vendor after contract termination	ex	0	ex	0	ex	0	ex	8−6
		Lack of interoperability between suppliers	ex	0	ex	0	ex	0	ex	8−6
project management Risk	RS.P	Change in the organizational environment that may affect the project stability	in	0	in	2−1	in	4−2	in	0
		Lack of commitment or the cooperation of the actors concerned	in	0	in	2−1	in	4−2	in	0
		Poor definition or permanent change of objectives	in	0	in	2−1	in	4−2	in	0
		Poor estimation of costs	in	0	in	1	in	1	in	0
		Poor estimation of maturities	in	0	in	1	in	1	in	0

## Appendix D. Measures of Theoretical Risk Estimation from the Case

**Table A9:** Measures of Theoretical Risk Estimation from the Case.

Type	Event	Uprooting			Construction			Stabilization			Optimization		
		Type	P(Ri)	I(Ri)	Type	P(Ri)	I(Ri)	Type	P(Ri)	I(Ri)	Type	P(Ri)	I(Ri)
Absence of personnel due to an accident	AB.P.1	ex	0	0	ex	1	2	ex	1	2	ex	1	1
	AB.P.2	in	1	2	in	1	2	in	1	2	in	1	1
Accidental lack or unavailability of service	AB.S.1	in	1	2	ex	1	2	ex	1	2	ex	1	2
	AB.S.2	in	1	1	ex	1	2	ex	1	2	ex	1	2
	AB.S.3	in	1	1	ex	1	1	ex	1	1	ex	1	1
	AB.S.4	in	0	0	in	0	0	ex	0	0	ex	1	2
	AB.S.5	in	0	0	in	0	0	ex	0	0	ex	1	2
Environmental serious accident	AC.E.1	ex	1	3	ex	1	2	ex	1	2	ex	1	2
	AC.E.2	ex	1	4	ex	1	3	ex	1	3	ex	1	3
	AC.E.3	ex	1	4	ex	1	3	ex	1	3	ex	1	3
Hardware Accident	AC.M.1	in	2	2	ex	1	2	ex	1	2	ex	1	2
	AC.M.2	in	3	1	ex	1	2	ex	1	2	ex	1	2
Voluntary absence of staff	AV.P.1	in	1	2	in	1	2	in	1	2	in	1	1
Design error	ER.L.1	in	1	2	in	1	2	ex/in	1	2	ex/in	1	1
Hardware error or behavioural error by personnel	ER.P.1	in	2	2	ex/in	2	1	ex/in	1	2	in	1	2
	ER.P.2	in	2	2	ex/in	2	1	ex/in	1	2	in	1	2
	ER.P.3	in	2	2	in	2	2	in	1	2	in	1	2
Incident due to environment	IC.E.1	in	1	2	in	1	2	ex/in	1	2	ex	1	2
	IC.E.2	in	1	2	in	2	2	ex/in	2	2	ex	2	2
	IC.E.3	in	2	3	in	2	2	ex/in	2	2	ex	2	2
	IC.E.4	in	1	2	in	1	2	ex/in	1	2	ex	1	2
Logical or functional incident	IF.L.1	in	0	0	in	1	1	in	1	1	in	1	1
	IF.L.2	in	2	2	in	2	1	ex/in	1	2	ex/in	1	2
	IF.L.3	ex	2	2	ex	1	1	ex	1	2	ex	1	2
	IF.L.4	ex	2	2	ex	1	1	ex	1	2	ex	1	2
Malevolent action (logical or functional)	MA.L.1	ex/in	0	0	ex	1	2	ex	1	3	ex	1	3
	MA.L.2	in	1	2	ex	1	2	ex	1	3	ex	1	3
	MA.L.3	in	1	2	ex/in	1	2	ex	1	3	ex	1	3
	MA.L.4	ex/in	1	1	ex	1	1	ex	1	2	ex	1	2
	MA.L.5	in	2	1	in	2	1	ex/in	2	2	ex/in	2	2
	MA.L.6	in	2	1	ex/in	2	1	ex/in	2	2	ex/in	2	2
	MA.L.7	in	2	1	ex	1	1	ex	1	2	ex	1	2
	MA.L.8	ex/in	2	1	ex	1	2	ex	1	2	ex	1	2
	MA.L.9	in	1	3	in	1	4	ex	1	4	ex	1	4
	MA.L.10	in	1	2	in	2	2	ex/in	2	3	ex/in	1	3
Malevolent action (physical)	MA.P.1	in	1	2	ex	1	2	ex	1	2	ex	1	2
	MA.P.2	ex/in	1	3	ex/in	1	3	ex/in	1	3	ex/in	1	3
	MA.P.3	ex/in	1	3	ex/in	1	3	ex/in	1	3	ex/in	1	3
	MA.P.4	ex/in	1	2	ex/in	1	2	ex/in	1	2	ex/in	1	1
Non compliance to procedures	PR.N.1	in	1	2	in	1	2	in	1	2	in	0	0
	PR.N.2	in	2	2	in	1	2	in	1	2	in	0	0
	PR.N.3	in	2	2	in	2	2	in	1	2	in	0	0
	PR.N.4	in	1	1	in	1	1	in	1	1	in	1	1
Cloud Computing Incident	IC.C.1	in	0	0	ex/in	2	2	ex/in	2	3	ex/in	1	2
	IC.C.2	ex	0	0	ex	1	2	ex	1	3	ex	1	3
	IC.C.3	ex	0	0	ex	0	0	ex	2	2	ex	3	3
	IC.C.4	ex	0	0	ex	0	0	ex	0	0	ex	2	3
	IC.C.5	ex	0	0	ex	0	0	ex	0	0	ex	2	3
project management Risk	RS.P.1	in	0	0	in	1	1	in	1	1	in	0	0
	RS.P.2	in	0	0	in	1	2	in	1	2	in	0	0
	RS.P.3	in	0	0	in	1	2	in	1	1	in	0	0
	RS.P.4	in	0	0	in	1	1	in	1	1	in	0	0
	RS.P.5	in	0	0	in	1	1	in	2	1	in	0	0

## References

[1] Wan J., Zhang D., Zhao S., Yang L., Lloret J. (2014). Context-aware vehicular cyber-physical systems with cloud support: Architecture, challenges, and solutions. IEEE Commun. Mag..

[2] Xu X. (2012). From cloud computing to cloud manufacturing. Robot. Comput.-Integr. Manuf..

[3] Troshani I., Rampersad G., Wickramasinghe N. Cloud Nine? An Integrative Risk Management Framework for Cloud Computing. Proceedings of the 24th Bled e Conference e Futere.

[4] Salleh S.M., Teoh S.Y., Chan C. Cloud Enterprise Systems: A Review of Literature and Its Adoption. Proceedings of the PACIS 2012.

[5] Abdul Rahman A.A.L., Islam S., Kalloniatis C., Gritzalis S. (2017). A Risk Management Approach for a Sustainable Cloud Migration. J. Risk Financ. Manag..

[6] Islam S., Fenz S., Weippl E., Mouratidis H. (2017). A risk management framework for cloud migration decision support. J. Risk Financ. Manag..

[7] Bouaynaya W. Mise en perspective théorique du construit sécurité dans le couplage Cloud Computing-Open Source. Proceedings of the 21ème Colloque de l’AIM.

[8] Prasad A., Green P., Heales J. On structural considerations for governing the cloud. Proceedings of the AMCIS 2013.

[9] Marston S., Li Z., Bandyopadhyay S. (2011). Cloud computing—The business perspective. Decis. Support Syst..

[10] Bhattacherjee A., Park S.C. (2014). Why end-users move to the cloud: A migration-theoretic analysis. Eur. J. Inf. Syst..

[11] Li Y., Chang K.C. A study on user acceptance of cloud computing: A multi-theoretical perspective. Proceedings of the AMCIS 2012.

[12] Nedbal D., Stieninger M., Erskine M. The Adoption of Cloud Services in the Context of Organizations: An examination of drivers and barriers. Proceedings of the AMCIS 2014.

[13] Cheng F.C., Lai W.H. (2012). The impact of cloud computing technology on legal infrastructure within internet—Focusing on the protection of information privacy. Procedia Eng..

[14] Subashini S., Kavitha V. (2011). A survey on security issues in service delivery models of cloud computing. J. Netw. Comput. Appl..

[15] Svantesson D., Clarke R. (2010). Privacy and consumer risks in cloud computing. Comput. Law Secur. Rev..

[16] Bernard J.G., Rivard S., Aubert B. (2016). L’exposition au risque d’implantation d’ERP: Éléments de mesure et d’atténuation. Syst. D’in. Manag..

[17] Barki H., Rivard S., Talbot J. (1993). Toward an assessment of software development risk. J. Manag. Inf. Syst..

[18] Schmidt R., Lyytinen K., Keil M. (2001). Identifying software project risks: An international Delphi study. J. Manag. Inf. Syst..

[19] Keil M., Tiwana A., Bush A. (2002). Reconciling user and project manager perceptions of IT project risk: A Delphi study 1. Inf. Syst. J..

[20] Addas S., Pinsonneault A. (2015). The many faces of information technology interruptions: A taxonomy and preliminary investigation of their performance effects. Inf. Syst. J..

[21] Stieninger M., Nedbal D., Wetzlinger W. (2014). Impacts on the organizational adoption of cloud computing: A reconceptualization of influencing factors. Procedia Technol..

[22] Armbrust M., Fox A., Griffith R. (2010). A view of cloud computing. Commun. ACM.

[23] Buyya R., Yeo C.S., Venugopal S. Market-oriented cloud computing: Vision, hype, and reality for delivering it services as computing utilities. Proceedings of the High Performance Computing and Communication (IEEE 2008).

[24] Koehler P., Anandasivam A., Dan M.A. Cloud services from a consumer perspective. Proceedings of the International Conference on Autonomous and Intelligent Systems (AIS 2010), Povoa de Varzim.

[25] Sultan N.A. (2011). Reaching for the “cloud”: How SMEs can manage. Int. J. Inf. Manag..

[26] Boehm B.W. (1991). Software risk management: Principles and practices. IEEE Softw..

[27] Linnerooth B.J., Wahlström B. (1991). Applications of Probabilistic Risk Assessments: The Selection of Appropriate Tools 1. Risk Anal..

[28] Aubert B.A., Bernard J.G. (2004). Mesure Intégrée du Risque Dans les Organisations.

[29] McCutcheon D., Stuart F.I. (2000). Issues in the choice of supplier alliance partners. J. Oper. Manag..

[30] Siponen M.T. (2005). Analysis of modern IS security development approaches: Towards the next generation of social and adaptable ISS methods. Inf. Organ..

[31] Siponen M., Baskerville R. A new paradigm for adding security into IS development methods. Proceedings of the Advances in Information Security Management & Small Systems Security.

[32] Organ J., Stapleton L. (2012). Information systems risk through a socio-technical lens: Future directions in systems risk research. IFAC Proc. Vol..

[33] Furnell S., Clarke N. (2012). Power to the people? The evolving recognition of human aspects of security. Comput. Secur..

[34] Organ J., Stapleton L. (2013). Information systems risk paradigms: Towards a new theory on systems risk. IFAC Proc. Vol..

[35] Coles-Kemp L. (2009). Information security management: An entangled research challenge. Inf. Secur. Tech. Rep..

[36] Ashenden D. (2008). Information Security management: A human challenge?. Inf. Secur. Tech. Rep..

[37] Dhillon G., Backhouse J. (2001). Current directions in IS security research: Towards socio-organizational perspectives. Inf. Syst. J..

[38] Becker J., Bailey E. A comparison of IT governance & control frameworks in cloud computing. Proceedings of the AMCIS 2014.

[39] Pettigrew A.M. (1997). What is a processual analysis. Scand. J. Manag..

[40] Besson P., Rowe F. (2011). Perspectives sur le phénomène de la transformation organisationnelle. Syst. D’in. Manag..

[41] Alter S., Sherer S.A. (2004). A general, but readily adaptable model of information system risk. Commun. Assoc. Inf. Syst..

[42] Barlette Y. (2008). Une étude des comportements liés à la sécurité des systèmes d’information en PME. Syst. D’in. Manag..

[43] Thiétart R.A. (2014). Méthodes de Recherche en Management.

[44] Tsoukas H., Chia R. (2002). On organizational becoming: Rethinking organizational change. Organ. Sci..

[45] Gersick C.J. (1988). Time and transition in work teams: Toward a new model of group development. Acad. Manag. J..

[46] Xavier M.G., Neves M.V., Rossi F.D. Performance evaluation of container-based virtualization for high performance computing environments. Proceedings of the 21st Euromicro International Conference on Parallel, Distributed and Network-Based Processing (PDP 2013).

[47] Bui T. (2015). Analysis of docker security. arXiv.

[48] Miles M.B., Huberman A.M. (2003). Analyse des Données Qualitatives.

[49] Gephart R.P. (2004). Qualitative research and the Academy of Management Journal. Acad. Manag. J..

